# The beta2 integrin CD11c distinguishes a subset of cytotoxic pulmonary T cells with potent antiviral effects in vitro and in vivo

**DOI:** 10.1186/1465-9921-6-70

**Published:** 2005-07-12

**Authors:** Marc Beyer, Hongwei Wang, Nina Peters, Sandra Doths, Cordula Koerner-Rettberg, Peter JM Openshaw, Jürgen Schwarze

**Affiliations:** 1 Department of Respiratory Medicine, NHLI, Imperial College London, Norfolk Place, London, UK; 2 Klinik für Kinder- und Jugendmedizin, St. Josef-Hospital, Ruhr-Universität Bochum, Alexandrinenstr. 3, 44791 Bochum, Germany

## Abstract

**Background:**

The integrin CD11c is known as a marker for dendritic cells and has recently been described on T cells following lymphotropic choriomeningitis virus infection, a systemic infection affecting a multitude of organs. Here, we characterise CD11c bearing T cells in a murine model of localised pulmonary infection with respiratory syncytial virus (RSV).

**Methods:**

Mice were infected intranasally with RSV and expression of β2 integrins and T lymphocyte activation markers were monitored by flow cytometry. On day 8 post RSV infection CD11c^+ ^CD8^+ ^and CD11c^- ^CD8^+ ^T cells were assessed for cytokine production, cytotoxic activity and migration. Expression of CD11c mRNA in CD8^+ ^T cells was assessed by quantitative PCR.

**Results:**

Following RSV infection CD11c^+ ^CD8^+ ^T cells were detectable in the lung from day 4 onwards and accounted for 45.9 ± 4.8% of CD8^+ ^T cells on day 8 post infection, while only few such cells were present in mediastinal lymph nodes, spleen and blood. While CD11c was virtually absent from CD8^+ ^T cells in the absence of RSV infection, its mRNA was expressed in CD8^+ ^T cells of both naïve and RSV infected mice. CD11c^+^, but not CD11c^-^, CD8^+ ^T cells showed signs of recent activation, including up-regulation of CD11a and expression of CD11b and CD69 and were recruited preferentially to the lung. In addition, CD11c^+ ^CD8^+ ^T cells were the major subset responsible for IFNγ production, induction of target cell apoptosis *in vitro *and reduction of viral titres *in vivo*.

**Conclusion:**

CD11c is a useful marker for detection and isolation of pulmonary antiviral cytotoxic T cells following RSV infection. It identifies a subset of activated, virus-specific, cytotoxic T cells that exhibit potent antiviral effects *in vivo*.

## Background

Beta2 integrins, which are restricted to leukocytes, consist of a common β-chain (CD18) and the distinct α-chains CD11a (LFA-1), CD11b (Mac-1/ CR3) or CD11c (p150,95/ CR4) [1]. While CD11a is expressed widely on leukocytes, expression of CD11b and CD11c was thought to be confined to cells of myeloid origin and these molecules have been used as markers to define certain cell populations, e.g. CD11b for macrophages [2] and CD11c for dendritic cells [3]. β_2 _integrins are of critical importance for the development of functional immune responses; a mutation in the CD18 gene, resulting in decreased expression of β_2 _integrins and in defective migration of granulocytes, causes an immune defect termed leukocyte-adhesion deficiency [1]. CD11a/LFA-1 has been shown to be involved in T cell activation [4], T cell recruitment [5] and target cell killing [6] by binding to its ligand intercellular adhesion molecule-1 (CD54) thus mediating adhesive cell-cell-interactions. Over the past ten years several groups have reported that CD11b is expressed on activated cytotoxic T cells [7,8] and that it is also involved in T cell migration into inflamed tissues [9]. CD11c expression on T cells has been detected initially on a population of intestinal intraepithelial lymphocytes [10] and more recently on CD8^+ ^T cells following systemic viral infections [11]. The studies regarding β_2 _integrins on T cells were mostly conducted in mouse-models of infection with LCMV, a virus that induces systemic immune responses involving many different organs [12]. Here, we studied CD11c^+ ^T cells during a localised infection of the lung with RSV, monitoring their distribution and comparing the pulmonary to the systemic immune response. We hypothesized that CD11c is a marker of antigen-specific cytotoxic T cells, which is expressed following activation, rather than being transferred from APC during cell-cell interactions. Such a transfer from APC to T cells has been described for the co-stimulatory molecule CD80 [13]. In addition, we sought to define a function of CD11c when expressed on T cells following RSV infection.

## Materials and methods

### Virus and animals

Human RSV, type A2 from ATCC (Rockville, MD) free of mycoplasma contamination was used. The virus was cultured on HEp-2 cells from ATCC in Dulbecco's modified Eagle Medium (Invitrogen, Paisley, UK) containing 5% heat inactivated fetal calf serum and 1 % Penicillin / Streptomycin both from Sigma (Gillingham, UK).

Female BALB/c AnNCrl mice, 8 to 12 weeks of age, free of specific pathogens, were obtained from Charles River Laboratories (Margate, UK) and kept under specific pathogen free conditions. All experimental animals used in this study were under a protocol approved by the Home Office, London, UK. Mice were infected under light anesthesia with isoflurane by intranasal inoculation of RSV (5 × 10^5 ^PFU in 70 μl). Controls were untreated or mock infected with RSV, inactivated by irradiation with UV-light. RSV infection was confirmed by plaque assay as described previously [14]. Infection could be demonstrated in all infected animals tested but not in controls.

### Experimental Protocols

Mice were infected with RSV. On days 0 (naive controls), 4, 6, 8, 10, and 21 post infection mice were sacrificed and lungs, spleens, mediastinal lymph nodes (MLN) and blood cells were harvested. For secondary RSV infection mice were infected 28 days after primary infection. Allergic airway inflammation was induced by intraperitoneal injection of OVA in Alum on days 0 and 14. On days 21 to 23 mice were challenged intranasally with 75 μl 1% OVA in PBS. Mice were sacrificed 24 hours after the last OVA challenge. Organs were disrupted using a steel mesh, and red blood cells were lysed with ACK lysis buffer. For isolation of CD8^+ ^cells lungs were minced, incubated in collagenase (Sigma) solution and mononuclear cells were prepared using a Ficoll (Biochrom) gradient (density = 1.09 g/l). Cell numbers were assessed by counting in a Neubauer chamber.

### Flow cytometry

Fc-receptors were blocked with anti-CD16/32-mAb (2.4G2), cells were incubated with appropriate antibodies for 20 min at 4°C, washed, and suspended in staining buffer. A FACScalibur flow cytometry cell analyzer (Becton Dickinson, Oxford, UK) was used for data acquisition and WinMDI software (Scripps institute, La Jolla, USA) for analysis. The following antibodies (Becton Dickinson) were used: anti-CD11c (HL3), anti-CD3 (145-2C11), anti-CD69 (H1.2F3), anti-CD54 (3E2), and anti-F4/80 (Serotec, Oxford, UK) were FITC-conjugated; anti-CD3 (145-2C11), anti-CD4 (RM4-5), anti-CD8 (53-6.7), anti-CD11b (M1/70), anti-CD11c (HL3), anti-CD40 (3/23), anti-CD62L (MEL-14), and anti-CD80 (16-10A1) were PE-conjugated; anti-CD8α (53-6.7) was CyChrome-conjugated; biotinylated anti-CD11c (N418, Serotec), anti-CD11a (M17/4), and anti-CD11c (HL3) were stained with streptavidin-CyChrome (Becton Dickinson). Rat IgG2a (R35-95), rat IgG2b (R35-38), mouse IgG2b (49.2) and hamster IgG (G235-2356) were used as isotype controls. RSV-M2-pentamers (ProImmune, Oxford, UK) were used according to manufacturer's instructions.

For intracellular cytokine staining, lung cell suspensions were cultured at a concentration of 10^6^/ml medium in 24 well plates for 4 h in the presence of PMA (50 ng/ml), ionomycin (500 ng/ml) (both Sigma, Gillingham, UK) and Golgi Plug (1 μl/ml) (Becton Dickinson). Following fixation and permeabilization cells were stained with anti-IFN γ (XMG1.2) or isotype control.

### Isolation of CD8^+ ^cells

Mononuclear cells from lungs were pooled and incubated with anti-CD8-coated magnetic beads from Miltenyi (Bergisch-Gladbach, Germany) according to manufacturer's instructions. Cells were sorted by Auto-MACS (Miltenyi). Purity of CD8^+ ^cells assessed by FACS was at least 90%. For sorting of CD8^+ ^cells into CD11c^+ ^and CD11c^- ^populations, cells were labeled with anti-CD11c-Fitc and anti CD8-PE-antibodies and sorted on a FACSDiva (Becton Dickinson).

### *In vitro *Cytotoxicity assay

The assay was performed as described [15]. Briefly, P815 cells (ATCC) were used as target cells and labeled with 2 μM RSV-M2-peptide (ABC Synthesis, London, UK), washed extensively and cultured in 96-well U-bottom plates (10^4 ^cells/well in 100 μl). Sorted populations of CD8^+^CD11c^+ ^or CD8^+^CD11c^- ^were labeled with CFSE (1 μM, Invitrogen, UK) and added to the target cells in the concentrations indicated, resulting in a final volume of 200 μl/well. In some experiments effector cells were pre-incubated with 5 μg/ml anti-CD11c-mAb (HL3; BD) or isotype control-mAb (20 minutes, 4°C) prior to co-culture with target cells. Cells were incubated for 4 hr (37°C, 95% CO_2_), washed, and incubated with PBS/BSA/0.1% sodium-azide containing 7-Aminoactinomycin D (7-AAD) (20 μg/ml, Sigma, UK). Afterwards cells were washed and fixed in 1% PFA containing Actinomycin D (10 μg/ml, Sigma, UK) and analyzed directly on a FACScalibur (BD). Staining of CFSE-negative cells for 7-AAD was analyzed and specific lysis calculated with the formula:

%specific lysis = 100 × (% 7-AAD^+ ^sample - % 7-AAD^+ ^basal)/(100 - %7-AAD^+ ^basal).

### *In vivo *Cytotoxicity assay

CD11c^+^CD8^+ ^or CD11c^-^CD8^+ ^cells isolated by FACSDiva (Becton Dickinson) from lungs harvested on dpi 8, were adoptively transferred (2.5 × 10^5 ^cells per recipient) by intra-tracheal application 1 hour after RSV infection of recipient mice. On dpi 4 RSV titers in the lungs were determined by plaque assay as described [14].

### Migration assay

Lungs of RSV infected mice were harvested on dpi 8, CD8^+ ^cells were isolated and labeled with 1 μM CFSE. Cells were incubated with 20 μg/ml of anti-CD11c antibodies (HL3, BD or N418, Serotec) or isotype controls for 30 minutes at 4°C and washed afterwards. Subsequently, cells were adoptively transferred by intravenous injection into recipient mice, which were either naïve or RSV infected (dpi4). After 3 or 24 hours lungs, perfused with PBS via the right ventricle, and spleens were harvested, cell suspensions were generated and analyzed by FACS.

### Real-time PCR

RNA was isolated from purified cells using RNeasy kit (Qiagen, Hilden, Germany) according to the manufacturer's instructions, including a step for on column DNA digestion. The isolated RNA was transcribed into cDNA using Omniscript kit (Qiagen). Primers and probes for CD11c (Applied Biosystems, Warrington, UK) and murine GAPDH (Qiagen) were used according to manufacturers' instructions. Samples were done as duplicates and analyzed on an ABI Prism 7000 light cycler PCR machine (Applied Biosystems).

### Statistical analysis

Data are expressed as mean ± standard deviation unless indicated otherwise. Groups were compared by students t-test. P values for significance were set at < 0.05.

## Results

### RSV infection results in increases in numbers of CD11c^+ ^CD8^+ ^T cells in the lung

**Figure 1:**
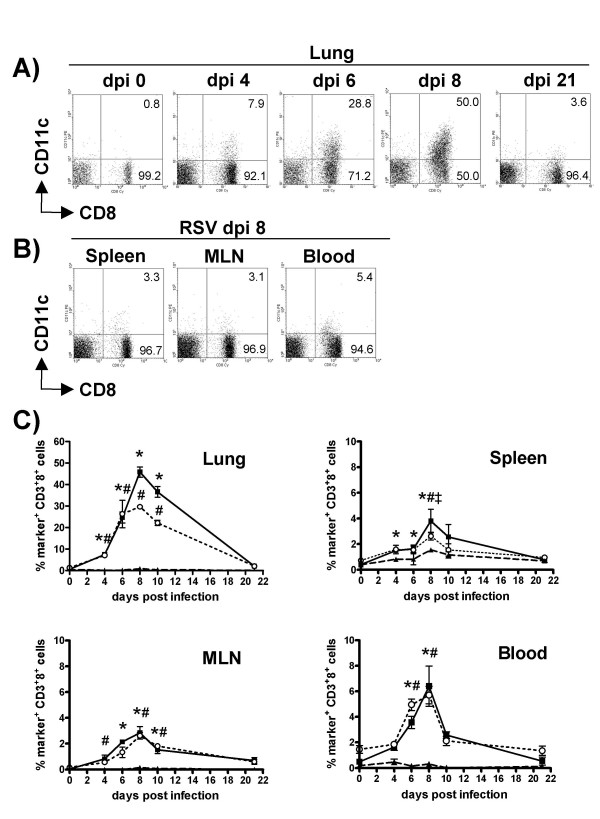
**Expression of CD11c on pulmonary CD8^+ ^T cells following RSV infection. **Following primary RSV infection lungs, spleens, MLN and blood were harvested from BALB/c mice at the time points indicated. Cells were stained with antibodies to CD3, CD8, and CD11c, CD11b or F4/80. Dot plots show expression of CD11c and CD8 on cells gated for CD3^+ ^lymphocytes harvested from A) lungs, B) spleen, MLN and blood. Numbers indicate the percentage of CD8^+ ^cells. Quadrant settings were adjusted that less than 1% of CD8^+ ^cells stained positive for the isotype control of anti-CD11c-mAb. C) Changes in expression of CD11c, CD11b and F4/80 on CD3^+ ^CD8^+ ^cells from lungs, spleen, MLN, and blood following RSV infection. Cells were stained with antibodies as indicated and analyzed by FACS. The percentage of CD3^+^8^+ ^cells staining positive for CD11c (black squares), CD11b (open circles) or F4/80 (triangles) after subtraction of isotype controls is shown as mean ± SEM (n = 6, *p < 0.05, significant difference versus dpi 0).

Recently, we have reported a population of CD8^+^CD11c^+ ^T cells in the lung ten days after RSV infection [14]. To study the kinetics of these cells during infection, BALB/c mice were infected with RSV, lungs were harvested at several time points after infection and CD3-positive lymphocytes were monitored for expression of CD11c by FACS. In naïve (Figure 1a, c) as well as mock-infected mice (data not shown) only as little as 0.7 ± 0.3% of pulmonary CD8^+ ^T cells expressed CD11c. Following RSV infection, numbers of CD11c^+ ^CD8^+ ^T cells increased significantly until dpi 8 when 45.9 ± 4.8% of CD8^+ ^T cells expressed CD11c and declined thereafter. Interestingly, expression of CD11c was hardly increased on CD3^+ ^CD8^- ^cells (Figure 1a), consisting mostly of CD4^+ ^T cells (data not shown).

To analyze if CD11c is also expressed on T cells in other organs than the lung during RSV infection, CD8^+ ^T cells were monitored in MLN, spleen and blood. In all of these organs small increases in CD11c^+ ^CD8^+ ^T cells were detected from dpi 4 onwards but their percentage returned to baseline rapidly. Only in MLN, numbers of CD8^+ ^T cells expressing CD11c remained significantly elevated on dpi 10 (Figure 1c). However, numbers of CD8^+ ^CD11c^+ ^cells in the lungs were 8 – 12-fold higher than in any other organ at any time point assessed after RSV infection. Next, we asked if expression of CD11c on CD8^+ ^T cells is unique to primary infection. Analysis of T cells from mice which were re-infected with RSV on dpi 28 showed that 31.9 ± 1.4% of pulmonary CD8^+ ^T cells were positive for CD11c already 4 days after secondary infection. Again CD11c expression was much lower in MLN, spleen or blood and was not increased on CD3^+^CD8^- ^cells (data not shown). Since both primary and secondary RSV infection resulted in appearance of CD8^+ ^CD11c^+ ^T cells we asked if CD11c expression on T cells is a general phenomenon in lung inflammation irrespective of etiology. To this end, allergic airway inflammation was induced in mice using a protocol, which leads to airway eosinophilia, as well as antigen-specific Th2 cytokine production by CD4^+ ^lung cells (data not shown). In this setting, only 3.8 ± 1.0% of CD8^+ ^T cells expressed CD11c and no changes were observed in the CD3^+ ^CD8^- ^population. To examine if other markers, expressed predominantly on cells of the myeloid lineage, are also increased on T cells during RSV infection we analyzed expression of CD11b and F4/80 on T cells from lungs, MLN, spleen and blood. Kinetics of CD11b expression on T cells paralleled CD11c expression, with lower percentages of CD11b^+ ^CD8^+ ^T cells in the lungs on dpi 8 and 10 (Figure 1c). Expression of the macrophage marker F4/80 was only detectable in significant amounts on splenic T cells on dpi 8 (Figure 1c).

### Activation markers are expressed differentially on CD11c^+ ^CD8^+ ^and CD11c^- ^CD8^+ ^T cells

**Figure 2:**
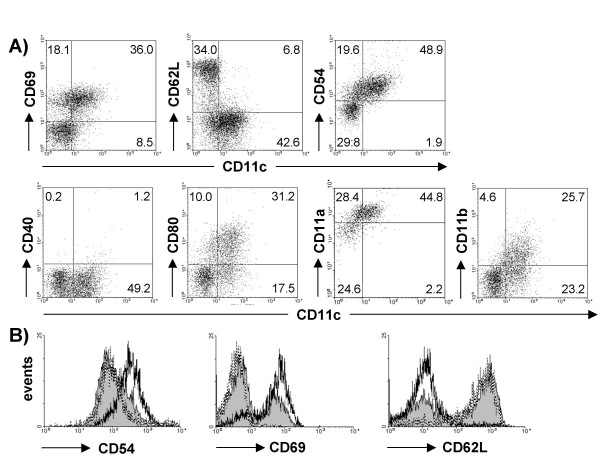
**Differential expression of surface markers on pulmonary CD11c^+ ^and CD11c^- ^CD8^+ ^T cells. **Lungs of naive mice or on day 8 after RSV infection were harvested. Cells were stained with antibodies for CD8, CD11c, and the surface markers indicated. A) Dot plots show expression of CD11c versus the indicated marker on cells gated for lymphocyte size and CD8 expression after RSV infection. Numbers indicate the percentage of cells in corresponding quadrants. Quadrants were set according to isotype controls except for CD11a, CD54 and CD62L. Since these three molecules are expressed to a considerable degree on naïve CD8 T cells, this expression was used for quadrant settings to highlight differences in CD11^+ ^CTL. B) Histograms show expression of CD54, CD62L, and CD69 on pulmonary CD8^+ ^cells from naïve (dotted line) or RSV infected mice (CD11c^+ ^cells: black line, CD11c^- ^cells: filled histogram).

Having observed striking increases of CD8^+ ^T cells expressing CD11c in the lung following RSV infection we asked if these cells are phenotypically different regarding their activation status compared to CD11c^- ^CD8^+ ^T cells. We analyzed pulmonary CD8^+ ^cells almost all of which are T cells (>99% CD3^+ ^in both naïve and infected mice) for surface markers known to be regulated during activation. CD69 was expressed on the majority of CD11c^+ ^T cells but only on a small proportion of CD11c^- ^T cells (Figure 2a). CD54, was up regulated on CD11c^+ ^T cells, while expression on CD11c^- ^T cells was comparable to pulmonary CD8^+ ^T cells from naïve mice (Figure 2b). CD62L is expressed at high levels on naïve and memory T cells and down-regulated on effector T cells. On most CD11c^+ ^T cells CD62L was detectable at low levels, while around two thirds of the CD8^+ ^CD11c^- ^population was CD62L^hi^. Further, while only a small proportion of CD8^+ ^T cells in MLN expressed CD11c, expression profiles of CD69 (CD11c^+ ^45.5 ± 2.3% versus CD11c^- ^7.7 ± 3.7%) and CD62L^high ^(CD11c^+ ^30.3 ± 8.5% versus CD11c^- ^87.1 ± 0.6%) on CD8^+ ^T cells (97% of CD11c^+ ^and 99% of CD11c^- ^cells were CD3^+^) indicated that these cells already begin to express CD11c following activation in the draining lymph nodes.

Since most activated CD8^+ ^T cells expressed CD11c, a surface molecule of myeloid cells including APC, we explored the expression of molecules involved in APC T cell-interaction on pulmonary CD8^+ ^T cells 8 days after RSV infection. While CD40 was detectable neither on CD11c^+ ^nor on CD11c^- ^CD8^+ ^T cells, expression of CD80 was confined mostly to CD11c^+ ^T cells (Figure 2a). In addition, we measured co-expression of the β_2 _integrins CD11c and CD11a or CD11b on CD8^+ ^T cells following RSV infection. Compared to CD11c^- ^CD8^+ ^T cells, CD11a was up regulated on CD11c^+ ^CD8^+ ^cells and CD11b was co-expressed on a subpopulation of CD11c^+ ^cells (Figure 2a).

### CD11c mRNA is expressed by both activated and resting CD8^+ ^T cells

Surface molecules such as CD80 can be transferred from APC to T cells by cell-cell contact [13]. Since both CD11c and CD80 are expressed widely on murine APC we asked whether the presence of CD11c on CD8^+ ^T cells is due to expression by T cells rather than to membrane transfer from APC during T cell priming. CD8^+ ^cells were purified from lungs and spleens of RSV-infected or naïve mice, RNA was isolated and the expression of CD11c analyzed by quantitative PCR. CD11c^+ ^pulmonary cells served as a positive control while LA-4 cells, a pulmonary epithelial cell line, and P815 cells, a mastocytoma cell line, were used as negative controls. In two separate experiments, expression levels of CD11c normalized to GAPDH were nearly equal in CD11c^+ ^(relative expression = 1.05 ± 0.06, n = 4) and CD8^+ ^(relative expression = 1.08 ± 0.03) cells from the lung following RSV infection. Surprisingly, CD11c mRNA could also be detected in comparable amounts in CD8^+ ^cells isolated from spleens of naïve mice (relative expression = 1.12 ± 0.08). CD11c mRNA was neither detectable in P815 cells nor in LA-4 cells. These findings suggest that CD11c mRNA is constitutively expressed in CD8^+ ^T cells.

### CD11c^+ ^CD8^+ ^cells are IFNγ producing, virus-specific, potent cytotoxic T cells

**Figure 3:**
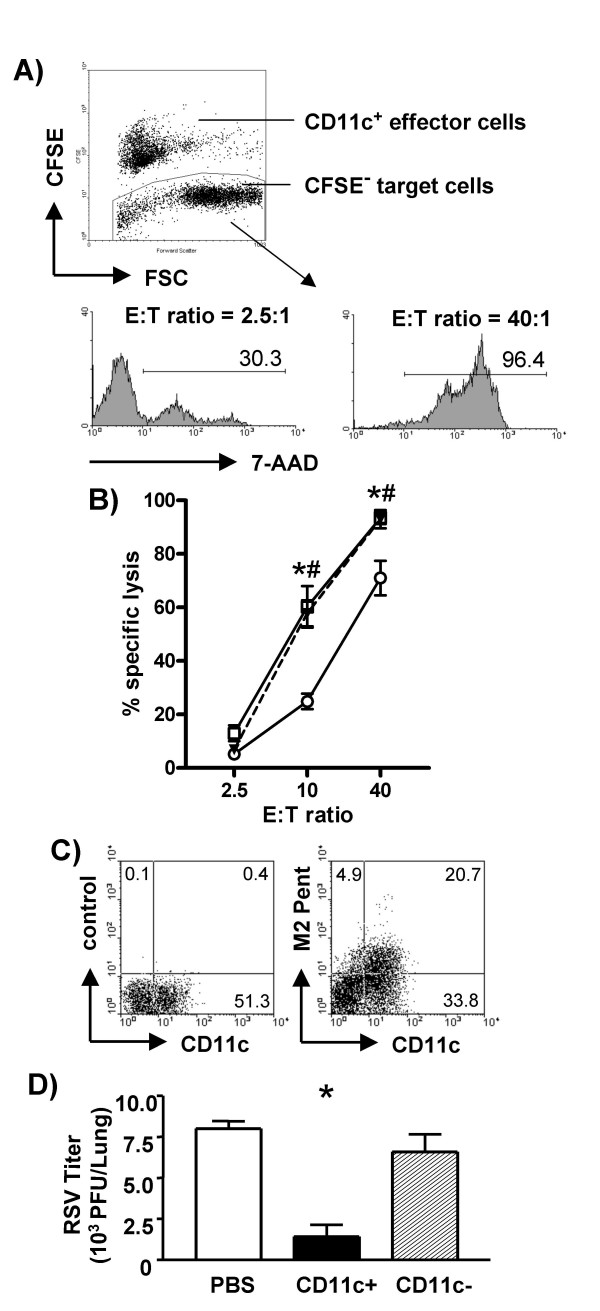
**Target cell lysis, binding of RSV M2-pentamer and reduction in viral titers by CD8^+ ^T cells following RSV infection. **Lungs were harvested on day 8 after RSV infection and CD8^+^11c^+ ^or CD8^+^11c^- ^cells were isolated. A) CFSE labeled effector and unlabeled P815 target cells were incubated at the indicated ratios for 4 hours. Subsequently the percentage of unlabeled cells, staining positive for 7-AAD, was determined by FACS and B) specific lysis was calculated (mean ± SD, n = 4, significant difference of * CD11c^+ ^(open squares) versus CD11c^- ^cells (open circles) or # anti-CD11c-mAb pretreated CD11c^+ ^(filled triangles) versus CD11c^- ^cells, p < 0.05) C) Freshly isolated lung cells were stained with anti-CD8, anti-CD11c and RSV M2-pentamer. Dot plots show expression of CD11c without or with RSV M2-pentamer staining on cells gated for CD8 expression. D) Isolated CD8^+^11c^+ ^or CD8^+^11c^- ^cells (2.5 × 10^5^) were adoptively transferred to the lungs 1 hour after RSV infection of recipients. Controls only received PBS intra-tracheally after infection. Lung RSV titers were determined by plaque assay on dpi 4. Depicted are mean ± SD from 2 independent experiments (n = 6, * significant difference, p < 0.05, CD11c^+ ^(black bar) versus PBS group (open bar) and CD11c^- ^cells (hatched bar).

To characterize the function of CD11c^+ ^T cells in RSV infection we determined their capacity to produce IFNγ. Pulmonary cell suspensions, harvested 8 days after RSV infection, were incubated with medium or PMA/ionomycin and CD8^+ ^T cells were analyzed for intracellular IFNγ production. In un-stimulated cells IFNγ was detectable in less than 3% of CD11c^+ ^or CD11c^- ^CD8^+ ^cells while following stimulation the percentage of IFNγ-producing CD11c^+ ^CD8^+ ^cells was significantly higher than in CD11c^- ^cells (38.4 ± 5.0% versus 25.5 ± 3.0%, respectively, n = 4, p < 0.05). The crucial function of CTL in viral infections is to induce apoptosis and lysis of infected cells to eliminate replicating virus. To determine if CD11c^+ ^T cells are more effective CTL during RSV infection than their CD11c^- ^counterparts we initially analyzed induction of apoptosis in RSV M2 peptide-labeled P815 cells. CD8^+ ^lung cells were isolated by MACS and enriched for CD11c^+ ^by FACS sorting, resulting in about 80% of CD11c^+ ^CTL in the positive population, while the negative fraction still contained about 25% of CD11c^+ ^CD8^+ ^T cells. These CD11c^+ ^and CD11c- CD8^+ ^T cell populations were used as effector cells in a cytotoxicity assay. Loss of membrane integrity in apoptotic CFSE^- ^target cells enables detection of dying cells by staining of DNA with the fluorescent dye 7-AAD (Figure 3a). CD11c^+ ^T cells were significantly more efficient in inducing apoptosis in target cells than CD11c^- ^T cells (Figure 3b). Pre-incubation of effector cells with anti-CD11c-mAb had no effect on the cytotoxic activity of either CD11c^+ ^(Figure 3b) or CD11c^- ^cells (data not shown). To assess the frequency of RSV-specific CTL directly we stained lung cells with RSV M2-pentamers, which recognize cells specific for this dominant CTL epitope. About 38% of CD11c^+ ^CTL were M2-Pentamer^+ ^and at 81% the majority of T cells expressing the RSV-M2 specific TCR was found to be CD11c^+ ^(Figure 3c). In contrast, only about 10% of CD11c^- ^CD8^+ ^T cells were specific for RSV M2-peptide. These results show that CD11c^+ ^CD8^+ ^T cells, which include the majority of RSV specific CTL, are highly effective in inducing target cell apoptosis *in vitro*. To compare antiviral efficiency of CD11c^+ ^and CD11c^- ^CD8^+ ^T cells *in vivo*, these cells were isolated by FACS sorting from lungs on dpi 8 and adoptively transferred one hour after RSV infection of recipient mice. Transfer of low numbers (2.5 × 10^5^) of CD11c^+ ^CTL (purity > 83%) significantly reduced lung RSV titers on dpi 4 (Figure 3d). Transfer of the same numbers of CD11c^- ^CD8^+ ^T cells (purity > 99%) did not have any effect, but transfer with tenfold higher numbers of CD11c^- ^CD8^+ ^T cells also reduced RSV titers in the lung significantly (data not shown).

### Preferential recruitment of CD11c^+ ^CD8^+^T cells to the lung

**Figure 4:**
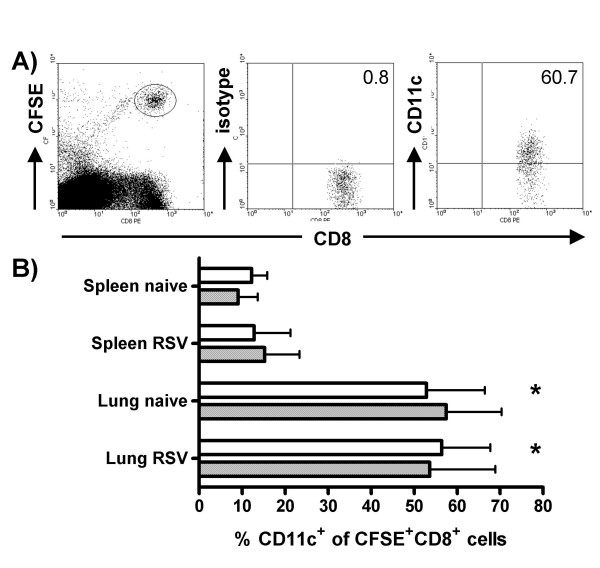
**Irrespective of infection, CD11c^+ ^T cells are recruited to the lung more effectively that to the spleen. **Lungs were harvested on day 8 after RSV infection; CD8^+ ^cells were isolated by magnetic cell sorting and labeled with CFSE. Subsequently cells were adoptively transferred by intravenous injection into naive or RSV infected mice. 3 hours after transfer lungs and spleens of recipient mice were harvested and expression of CD11c on transferred cells was analyzed by FACS. A) Dot plots show gating of transferred CFSE^+^CD8^+ ^cells and staining of gated cells with isotype control or anti-CD11c antibody. B) The bars indicate percentage of transferred CSFE^+ ^CD8^+ ^cells staining positive for CD11c in lungs and spleens after pre-incubation of transferred cells with isotype control (open bars) or anti-CD11c-mAb (hatched bars) (mean ± SD, n = 4, * significant difference, p < 0.05, lung naïve and lung RSV versus spleen naïve and spleen RSV).

As CD11c expression is associated with activation of virus-specific T cells but does not seem to have a specific, non-redundant function in cytotoxic activity, we asked if CD11c may be involved in migration of these cells to the lung. CD8^+ ^cells containing 49.9 ± 1.2 % CD11c^+ ^cells were isolated from lungs of mice 8 days after RSV infection, labeled with CFSE and injected intravenously into naïve mice, to assess their migratory behavior. Lungs and spleens of recipient mice were harvested 3 hours after cell transfer and the percentage of CD11c^+ ^cells in the population of CFSE^+ ^CD8^+ ^cells, was analyzed by FACS (Figure 4a). In lungs of naïve mice 52.9 ± 13.6% of transferred CD8^+ ^cells were CD11c^+^, while only 12.2 ± 3.6% stained positive for CD11c in the spleen. Total numbers of CD11c^+ ^CD8^+ ^T cells migrating to the lung (10.4 ± 8.4 × 10^3^, n = 5, p < 0.05) were also significantly higher than those reaching the spleen (6.3 ± 4.5 × 10^3^) in naïve mice. While the percentages of CD11c^+ ^cells of CFSE labeled CD8^+ ^T cells in the organs did not change significantly when cells were transferred into RSV infected mice (Figure 4b), absolute numbers of CD11c^+ ^CD8^+ ^T cells migrating to the lung increased significantly to 20.0 ± 7.2 × 10^3^, (n = 5, p < 0.05). In contrast, migration to the spleen did not change significantly if recipients were RSV infected (2.9 ± 2.8 × 10^3^). Further, no significant changes were observed when cells were harvested 24 instead of 3 hours after cell transfer (data not shown). Pre-incubation of CD8^+ ^cells with two different anti-CD11c-mAbs (clone HL3 or clone N418) did not affect recruitment into the lung.

## Discussion

Expression of β_2 _integrins on T cells following viral infections has been described in lymphatic organs [7,8,11] but knowledge about integrin expression on T cells following respiratory infections is scarce. Previously, we have described a population of CD8^+ ^T cells in the lung expressing the β_2 _integrin CD11c following RSV infection [14]. To characterize these cells in more detail, we have performed a time course analysis of CD3^+ ^cells displaying CD11c, thus excluding cells of myeloid origin. In mediastinal lymph nodes, where CD11c^+ ^cell numbers increase during inflammation [17,18], CD8α expressing dendritic cells [19,20] could be mistaken for CD11c^+ ^CD8^+ ^T cells if analyzed based on CD11c and CD8α only. Following RSV infection, CD11c expression on lung CD8^+ ^T cells increased with up to 50% of CD8 T cells positive for CD11c on dpi8, while, in contrast to LCMV infection, percentages of CD11c^+ ^CTL in spleen and blood were low. CD11c up-regulation on mucosal T cells has previously only been described in the intestine where a subpopulation of intraepithelial T cells expresses CD11c constitutively [10]. In the lung, expression of CD11c on T cells was restricted to CD8^+ ^T cells and was paralleled by increases in CD11b. In contrast to CD11b and CD11c, expression of F4/80 was hardly detectable on pulmonary T cells following RSV infection. Expression of F4/80 may be dependent on both mouse strain and organ analyzed: F4/80^+ ^T cells seem to be more prevalent in C57Bl/6 than in BALB/c mice [11] and were detectable in significant amounts only on splenic CD8^+ ^T cells, as shown here and by Lin and colleagues [11]. In contrast to viral infections, allergic airway inflammation elicited by sensitization to OVA, resulted only in a small population of CD11c^+ ^CD8^+ ^T cells in the lung, demonstrating that up-regulation of CD11c is not a consequence of T cell activation and migration per se or of an inflammatory environment, but that it is dependent on the underlying pathology. Interestingly, reactivation of antigen-specific CD8^+ ^memory T cells by OVA results in CD11c up-regulation when cells are primed with an OVA-expressing virus [21]. This suggests that MHC class I-restricted antigen presentation as well as co-stimulatory factors induced by viral infection, e.g. IFNα, favor expression of CD11c on CD8^+ ^T cells. Several studies have demonstrated up-regulation of CD11a [22], expression of CD11b [7,8,23] or CD11c [11] on T cells following viral infections. Here, we demonstrate that following RSV infection a population of CTL co-expresses CD11a^hi^, CD11b and CD11c. Furthermore CD11c mRNA was detectable in resting as well as activated CD8^+ ^T cells. Contamination by CD11c^+ ^non-T cells cannot be excluded completely but is very unlikely to account for CD11c mRNA detection in CD8 T cells since no major difference in CD11c mRNA quantity was detected between CD8^+ ^and CD11c^+ ^cell populations. CD11c mRNA seems to be expressed constitutively in CD8^+ ^T cells while expression of CD11c protein on the cell surface requires activation. This notion is supported further by the finding that activation markers were up regulated on CD11c^+ ^T cells both in the lung and in the draining MLN. Interestingly, CD11b mRNA is also expressed in resting as well as activated human T cells but CD11b becomes detectable on the cell surface only following stimulation of T cells [24]. Taken together these observations make it tempting to speculate that there may be a common mechanism of post-transcriptional regulation of β_2 _integrin expression following T cell activation.

Assessing functional properties of CD11c^+ ^CD8^+ ^T cells, we found that these were more efficient than CD11c^- ^CD8^+ ^T cells in IFNγ production, target cell lysis *in vitro *and induction of viral clearance *in vivo*. This is likely due to the fact that the CD11c^+ ^CTL population contains the majority of activated T cells specific for the RSV M2_82–90_-peptide, the immuno-dominant CTL epitope of RSV in BALB/c mice. Thus, we show an association of adhesion molecule expression and high cytotoxic activity of pulmonary CD8^+ ^T cells during a respiratory viral infection, parallel to findings in lymphoid organs in LCMV infection [8,11]. A subset of CD11c^- ^CD8^+ ^T cells expressed high levels of the activation marker CD69 and low levels of CD62L. Cytotoxic effects induced by CD11c^- ^CD8^+ ^cells could be due to this subpopulation of activated CD11c^- ^CTL. We cannot exclude this, since we did not assess cytotoxic effects of isolated subpopulations of CD11c^- ^CD8^+ ^T cells. We believe though, that contamination with CD11c^+ ^CTL is a more likely explanation for cytotoxic activity observed when CD11c^- ^cells were used as effectors. In contrast to a study implicating CD11c on human T cell lines in target cell binding [25] we could not detect an inhibitory effect of anti-CD11c-mAb on the cytotoxic activity of T cells. In addition to target cell binding, β_2 _integrins are involved in migration of leukocytes and a role for CD11b in recruitment of T cells into inflamed areas has been shown by antibody blocking experiments [9]. In our model, the percentage of CD11c^+ ^cells within the CD8^+ ^T cell population differed between the organs assessed, being lowest in MLN and spleen, higher in the blood, where a short-lived increase of these cells was noted during the acute phase of infection, and highest in the lung. This indicates that CD11c marks effector T cells, which are recruited to the site of infection. An analogous pattern of tissue distribution has been described for VLA-4^hi ^T cells following intra-cerebral LCMV infection [8]. Interestingly, following adoptive transfer we observed efficient recruitment of CD11c^+ ^CD8^+ ^T cells to the lung in naïve mice, while only a small percentage of these cells were recruited to the spleen. This preferential recruitment of CD11c^+ ^CD8^+ ^T cells to the lung was even more pronounced in RSV infected recipients. Pre-treatment of transferred cells with anti-CD11c-mAb did not reveal a unique function of CD11c for homing of activated CTL to the lung. These observations suggest that CD11c is a marker for activated CTL, which are recruited preferentially to the lung, while the CD11c molecule itself may not be directly involved in the process of T cell recruitment to the lung. Functional redundancy of β_2 _integrins in target cell binding or migration into effector sites is a possible explanation of our results. CD11a has been shown to be important for target cell binding [26], recruitment of activated lymphocytes into the lung [5,27] and generation of an effective cytotoxic T cell response during primary RSV infection [28].

## Conclusion

We demonstrate that the β_2 _integrin, CD11c, identifies a subset of activated, virus-specific, cytotoxic CD8^+ ^T cells. These cells are preferentially recruited to the lung in RSV infection and exhibit potent antiviral effects *in vivo*. CD11c is expressed by CD8^+ ^T cells, but this molecule may not be directly involved in effector functions. Thus, following respiratory viral infections CD11c is a useful marker for detection and isolation of activated, cytotoxic, pulmonary T cells, provided distinction from myeloid cells is taken into account.

## Abbreviations

7-AAD 7-Aminoactinomycin D

APC antigen presenting cells

CFSE 5-(and-6)-carboxyfluorescein diacetate, succinimidyl ester

CTL cytotoxic T lymphocytes

GAPDH Glycerol aldehyde phosphate dehydrogenase

IFN interferon

LCMV lymphotropic choriomeningitis virus

MHC major histocompatibility complex

MLN mediastinal lymph nodes

OVA chicken ovalbumin

RSV respiratory syncytial virus

## Authors' contributions

MB: Experimental design, cell isolation, cytotoxicity assay, adoptive cell transfers and flow cytometry, preparation of manuscript

HW: Designed and conducted experiment assessing viral clearance after T cell transfers.

NP: Quantitative PCR and adoptive cell transfers

SD: Animal infection, cell isolation, flow cytometry

CK-R: Cell isolation, flow cytometry

PJO: Experimental design

JS: Experimental design, preparation of manuscript

All authors have read and approved the final manuscript.
